# Gallstone Pancreatitis Post Laparoscopic Cholecystectomy: A Case Report

**DOI:** 10.7759/cureus.39704

**Published:** 2023-05-30

**Authors:** Jonathon H Hines, Sujesh Pillai

**Affiliations:** 1 College of Osteopathic Medicine, Sam Houston State University, Conroe, USA; 2 Department of Internal Medicine, Huntsville Memorial Hospital, Huntsville, USA

**Keywords:** cholecystectomy, magnetic resonance cholangiopancreatography (mrcp), endoscopic retrograde cholangiopancreatography (ercp), biliary pancreatitis, gallstone pancreatitis

## Abstract

Gallstone pancreatitis is uncommon after laparoscopic cholecystectomy with minimal cases reported in the literature. We report a case of a 38-year-old female who developed gallstone pancreatitis three weeks after laparoscopic cholecystectomy. The patient presented to the emergency department with a two-day history of severe right upper quadrant and epigastric pain radiating to her back with associated nausea and vomiting. The patient had elevated total bilirubin, aspartate aminotransferase (AST), alanine aminotransferase (ALT), alkaline phosphatase (ALP), and lipase. The patient’s preoperative abdominal magnetic resonance imaging (MRI) and magnetic resonance cholangiopancreatography (MRCP), prior to her cholecystectomy, were negative for common bile duct stones. However, it is important to note that common bile duct stones are not always visible on ultrasound, MRI, and MRCP prior to cholecystectomy. In our patient, an endoscopic retrograde cholangiopancreatography (ERCP) revealed gallstones in the distal common bile duct, which were removed with biliary sphincterotomy. The patient had an uneventful postoperative recovery. It is important for physicians to have a high index of suspicion for gallstone pancreatitis in a patient with epigastric pain radiating to the back with a known history of recent cholecystectomy, as this is a diagnosis that can be missed due to its infrequent occurrence.

## Introduction

Gallstone pancreatitis is a well-documented phenomenon in the literature. However, nearly all the published literature is prior to any surgical intervention. Gallstone pancreatitis after laparoscopic cholecystectomy is rare because common bile duct stones are almost always found prior to surgery. Symptomatic retained gallstones after laparoscopic cholecystectomy are an uncommon occurrence with an estimated prevalence of approximately 2-3% [1]. Here, we present an infrequent case of a patient with signs and symptoms of gallstone pancreatitis three weeks after a laparoscopic cholecystectomy that was subsequently found to have a retained gallstone in her distal common bile duct during endoscopic retrograde cholangiopancreatography (ERCP).

## Case presentation

Our patient was a 38-year-old overweight Hispanic female status three weeks post-cholecystectomy. She initially presented to the emergency department with a four-day history of nausea, vomiting, and progressively worsening right upper quadrant pain exacerbated by eating. She had a known history of asymptomatic cholelithiasis for the past year. Labs were drawn and demonstrated elevated alanine aminotransferase (ALT) (607 U/L), aspartate aminotransferase (AST) (532 U/L), amylase (728 U/L), and lipase (> 1,200 U/L) raising suspicion for choledocholithiasis. A gallbladder ultrasound was obtained showing cholelithiasis with sludge and a dilated common bile duct at 12 mm. With ultrasound, a filling defect was visualized in the distal common bile duct. An abdominal MRI was ordered and showed multiple gallstones within the gallbladder and a mildly dilated common bile duct at 9 mm. MRI also showed mild edema along the pancreas concerning for acute pancreatitis. Surgery was consulted and deferred evaluation of the common bile duct to gastroenterology who opted to perform magnetic resonance cholangiopancreatography (MRCP), which showed multiple gallstones in the gallbladder and a mildly dilated common bile duct with no evidence of choledocholithiasis. At that time, gastroenterology stated the common bile duct dilation was likely related to pancreas inflammation or a stone that had already passed because no common bile duct stones were found during MRCP. After the negative MRCP, the patient was then taken to surgery where a laparoscopic cholecystectomy was performed without complications. The patient recovered well and was discharged the next day.

**Figure 1:**
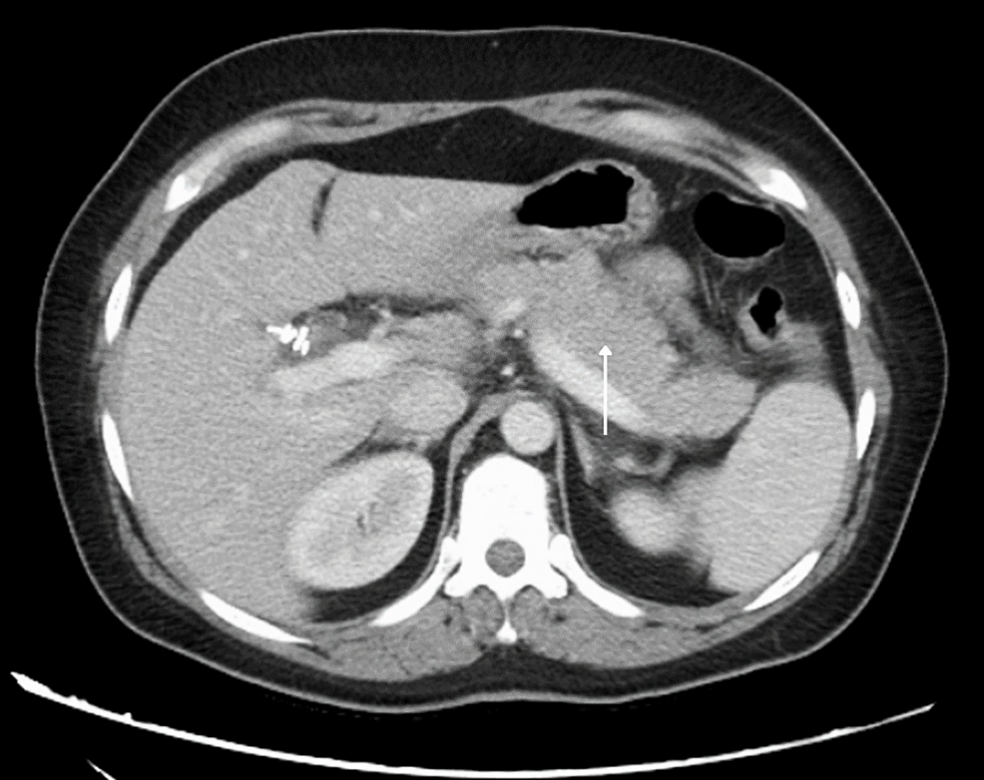
Preoperative computed tomography demonstrating an edematous, inflamed pancreas consistent with acute pancreatitis.

Three weeks after the cholecystectomy, the patient presented to the emergency department again with a two-day history of nausea, vomiting, and severe abdominal pain radiating to her back. She denied fever, chills, jaundice, and changes in her bowel habits. At the time of presentation to the emergency room, the patient was taking no home medications other than ondansetron 4 mg as needed for nausea. Past medical history was negative for any chronic medical conditions. The patient denied any current or past use of tobacco, alcohol, or illicit drugs. Family history was non-contributory. On physical exam, her abdomen was soft with marked tenderness in her right upper quadrant and epigastric regions with mild abdominal distention and hypoactive bowel sounds. Her white blood cell count was 6.4 x 103/uL and hemoglobin was 10.2 g/dL. Liver function tests (LFTs) revealed elevated total bilirubin, AST, ALT, and ALP as shown in Table 1. Abdominal computed tomography (CT) was ordered and demonstrated hepatomegaly attributed to body habitus and a mildly edematous pancreas without evidence of choledocholithiasis or common bile duct dilation (Figure 1).

**Table 1:** Patient laboratory values demonstrating downward trend during three-day hospitalization. AST: aspartate aminotransferase, ALT: alanine aminotransferase, ALP: alkaline phosphatase

	November 27, 2022	November 28, 2022	November 29, 2022	Reference Range
Total Bilirubin (mg/dL)	1.5	0.4	0.4	0.1 – 1.0 mg/dL
AST (U/L)	577	144	50	12 – 38 U/L
ALT (U/L)	457	303	203	10 – 40 U/L
ALP (U/L)	187	176	149	25 – 100 U/L
Lipase (U/L)	> 1,200			0 – 160 U/L

**Figure 2:**
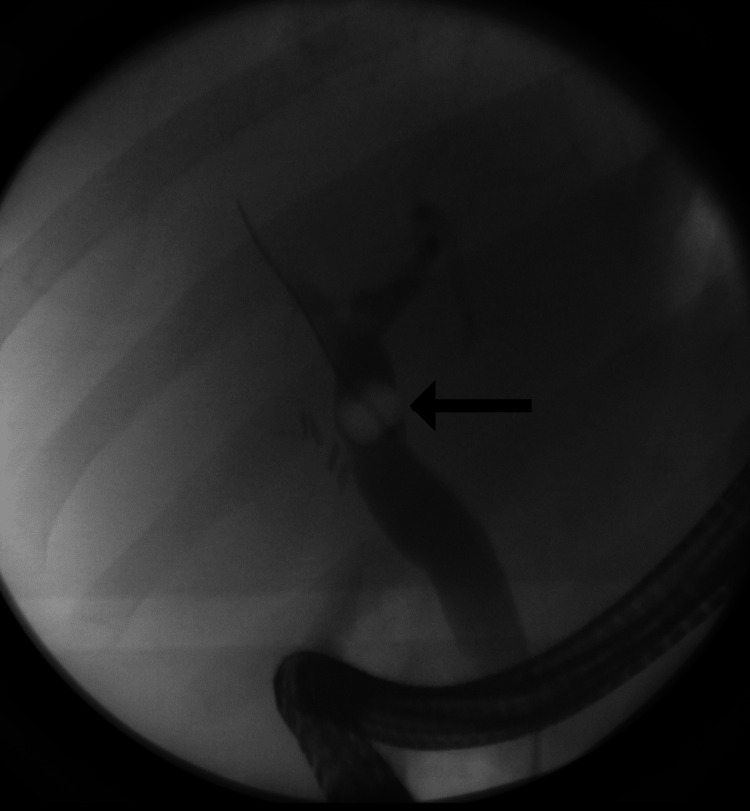
Endoscopic retrograde cholangiopancreatography revealing a gallstone lodged in the common bile duct.

Due to the patient’s symptoms and corresponding labs, gallstone pancreatitis was at the top of the differential and a decision was made to consult gastroenterology to perform an ERCP. This procedure was proposed to the patient and her husband who agreed and provided informed consent. ERCP showed several small filling defects in the distal common bile duct, a common bile duct stone, and a small amount of papillary stenosis (Figure 2). A biliary sphincterotomy was then performed with balloon retrieval of stones and sludge. After the removal of the balloon, there was a good flow of bile and contrast through the sphincterotomy into the duodenum. The final contrasted cholangiogram did not demonstrate any filling defects. The patient’s postoperative recovery was uneventful.

On hospital day two, the patient was feeling much better with slight tenderness remaining in her right upper quadrant and epigastric regions with accompanying nausea. The patient’s laboratory values were all trending down toward baseline including her total bilirubin, AST, ALT, and ALP. These lab values continued their downward trend on hospital day three as evidenced previously by Table 1, and the patient was subsequently discharged. 

## Discussion

Cholelithiasis is when one or more calculi (gallstones) are present in the gallbladder and is a common occurrence with an estimated prevalence of 10-20% in the United States [2]. Traditional risk factors for the production of cholesterol gallstones include age, female sex, obesity, and pregnancy [3]. Most gallstones are asymptomatic and do not progress to cholecystitis, which is when the gallbladder becomes inflamed or infected due to a stone becoming stuck and impacted in the cystic duct blocking the passage of bile.

Patients with cholecystitis classically present with biliary colic, which causes a sudden onset of severe, intermittent, right upper quadrant pain often lasting for more than 30 minutes at a time. Patients may also have accompanying nausea and vomiting or pain in the epigastrium, back, or right shoulder. These symptoms are often postprandial occurring after a meal heavy in fat and cholesterol which encourages the production of bile that cannot move past the cystic duct impaction. Physical exam maneuvers including Murphy’s Sign can help a physician narrow their differential by pressing in the right upper quadrant while the patient breathes in. Inspiration causes the diaphragm to move inferiorly and compress the abdominal contents causing the patient to quickly stop breathing to avoid pain. Murphy’s Sign is not a perfect test but is a useful tool in making a diagnosis of cholecystitis. However, clinicians should be aware that this exam is less accurate in older populations [4].

The cystic duct emanating from the gallbladder is small and tortuous, which is why the majority of gallstones do not reach the common bile duct, resulting in choledocholithiasis or gallstone pancreatitis. Gallstone pancreatitis, also known as biliary pancreatitis, occurs when one or more gallstones get lodged in the common bile duct distal to the main pancreatic duct causing the pancreas to become inflamed due to either the blockage of pancreatic digestive enzymes being released or reflux of bile into the main pancreatic duct. There are certain risk factors that can cause cholelithiasis to progress to gallstone pancreatitis. These risk factors include the patient having multiple gallstones and gallstones that are very small. The more gallstones a patient has, the greater their chances are of one of those stones being ejected into the biliary tract. Due to the small width of the cystic duct and its tortuous nature, it makes sense that small gallstones can traverse the cystic duct and are more likely to become lodged in the common bile duct causing choledocholithiasis or in the Ampulla of Vater causing gallstone pancreatitis. Therefore, it is not surprising that patients who have three or more stones, or stones smaller than 7 mm, are at significantly higher risk [5].

Symptoms of gallstone pancreatitis include epigastric pain that radiates to the back. This pain is often experienced posteriorly as well because the body and tail of the pancreas reside in the retroperitoneum of the abdomen. Patients often have accompanying nausea and vomiting. Gallstone pancreatitis has lab findings that help clue the diagnostician into the diagnosis including elevated ALT, AST, ALP, and lipase as seen in this patient. Typically, ALT will be elevated more than AST. ALT levels greater than 60 U/L in nonobese patients without a medical history of heavy drinking of alcoholic beverages can indicate pancreatitis with a biliary origin [6].

Cholelithiasis and gallstone pancreatitis can occur simultaneously and, when symptomatic, warrant a procedure known as a cholecystectomy in which the gallbladder is surgically removed from the body. This is typically done laparoscopically or robotically but can require an open procedure if complications occur.

Gallstones in the gallbladder can be visualized with ultrasound, while gallstones in the common bile duct often warrant either MRCP or ERCP to be fully visualized. MRCP is simply a diagnostic option that allows for the visualization of the common bile duct while ERCP takes this a step further allowing for an immediate therapeutic intervention in addition to visualization for bile duct stones [7]. Common bile duct stones are almost always found prior to a cholecystectomy, which is why it is unusual that our patient did not show any evidence on imaging prior to her gallbladder removal. Due to a high clinical suspicion for retained gallstones despite no evidence on CT or MRCP, we consulted Gastroenterology who agreed that an ERCP was appropriate for our patient. During an ERCP, retained gallstones can be easily visualized and removed via a biliary sphincterotomy in which the opening of the Ampulla of Vater is widened to allow retrieval or passage of the stones and sludge. While ERCP does provide an immediate diagnostic and therapeutic treatment option, it is important to note that it has a higher rate of complications than MRCP around 5-6% with pancreatitis being the most frequent adverse event after an ERCP [8].

Symptomatic retained gallstones after laparoscopic cholecystectomy are an uncommon occurrence with an estimated prevalence of 2-3% [1]. Patients should be made aware of known complications that may occur due to choledocholithiasis after laparoscopic cholecystectomy. A retrospective chart review by Spataro et al. described a single cohort of 100 patients with a known complication due to choledocholithiasis post cholecystectomy. In this study, the most common complication present in the cohort was biliary colic at 62%, acute cholangitis at 32%, and biliary pancreatitis at 6% [9]. Additional research with a larger population would need to be performed to confirm the incidence of complications due to choledocholithiasis post cholecystectomy as this sample population already presented with a complication and is not representative of a typical sample population cholecystectomy, which would include those without complications and thus lower these percentages. Literature also suggests that bile duct injury or a bleeding vessel are common complications in the early postoperative period and most cases of acute pancreatitis post cholecystectomy are transient and self-limiting unless there is an obstruction in the lower common bile duct [10,11]. In rare occasions, patients can develop post-cholecystectomy syndrome, which can manifest for months to years after a cholecystectomy resulting in right upper quadrant or epigastric pain after meals, jaundice, dyspepsia, and diarrhea.

Here, we reported a unique case of a patient with one of these rare occurrences to educate the community about how these patients with retained gallstones may present and to provide an example of a diagnostic and therapeutic plan of action for future patients. It is important to keep gallstone pancreatitis in the differential diagnosis for a patient after a cholecystectomy even if prior imaging was negative for stones in the common bile duct as was the case with this patient. A high level of suspicion and a broad differential are essential when investigating a patient’s problems, taking care not to rule things out of the differential too early because of previous procedures.

## Conclusions

Gallstone pancreatitis is an uncommon finding after a cholecystectomy. The diagnosis should be considered in a patient with epigastric pain radiating to the back with imaging consistent with acute pancreatitis. The patient will also have elevated aminotransferases and lipase. These findings after a cholecystectomy should raise the physician’s index of suspicion for gallstone pancreatitis from a gallstone retained in the common bile duct. This case report demonstrates how a patient with gallstone pancreatitis would likely present after a cholecystectomy and how ERCP can provide a definitive diagnosis as well an effective therapeutic intervention for these patients.
